# A study of brain functional network and alertness changes in temporal lobe epilepsy with and without *focal to bilateral tonic–clonic seizures*

**DOI:** 10.1186/s12883-021-02525-w

**Published:** 2022-01-07

**Authors:** Liluo Nie, Yanchun Jiang, Zongxia Lv, Xiaomin Pang, Xiulin Liang, Weiwei Chang, Jinou Zheng

**Affiliations:** Department of Neurology, the First Affiliated Hospital of Guangxi Medical University, No.6, Shuangyong Road, Nanning, 530021 China

**Keywords:** Temporal lobe epilepsy, Graph theory, Functional networks, Focal to bilateral tonic-clonic seizures, Alertness

## Abstract

**Background:**

Temporal lobe epilepsy (TLE) is commonly refractory. Epilepsy surgery is an effective treatment strategy for refractory epilepsy, but patients with a history of focal to bilateral tonic-clonic seizures (FBTCS) have poor outcomes. Previous network studies on epilepsy have found that TLE and idiopathic generalized epilepsy with generalized tonic-clonic seizures (IGE-GTCS) showed altered global and nodal topological properties. Alertness deficits also were found in TLE. However, FBTCS is a common type of seizure in TLE, and the implications for alertness as well as the topological rearrangements associated with this seizure type are not well understood.

**Methods:**

We obtained rs-fMRI data and collected the neuropsychological assessment data from 21 TLE patients with FBTCS (TLE- FBTCS), 18 TLE patients without FBTCS (TLE-non- FBTCS) and 22 controls, and constructed their respective functional brain networks. The topological properties were analyzed using the graph theoretical approach and correlations between altered topological properties and alertness were analyzed.

**Results:**

We found that TLE-FBTCS patients showed more serious impairment in alertness effect, intrinsic alertness and phasic alertness than the patients with TLE-non-FBTCS. They also showed significantly higher small-worldness, normalized clustering coefficient (γ) and a trend of higher global network efficiency (gE) compared to TLE-non-FBTCS patients. The gE showed a significant negative correlation with intrinsic alertness for TLE-non-FBTCS patients.

**Conclusion:**

Our findings show different impairments in brain network information integration, segregation and alertness between the patients with TLE-FBTCS and TLE-non-FBTCS, demonstrating that impairments of the brain network may underlie the disruptions in alertness functions.

**Supplementary Information:**

The online version contains supplementary material available at 10.1186/s12883-021-02525-w.

## Background

Temporal lobe epilepsy (TLE) is the most common type of focal epilepsy in adults, originating from the structure of the temporal lobe and propagating throughout brain networks that interconnect within the temporal lobe or extratemporal regions [1–3]. While TLE is often pharmaco-resistant in adults, surgery can be effective for resolving drug-tolerant epilepsy. Although about 60% of patients remain seizure-free post-operation [4], 30-40% of patients can relapse within 2 years after surgery [5]. A history of focal to bilateral tonic-clonic seizures (FBTCS)may predict a poorer seizure outcome compared to TLE alone [6, 7]. The prevalence of FBTCS is associated with poorer quality of life, additional risk of fatal injuries related to seizure and unfavorable postoperative outcomes [5, 8]. Why some TLE patients with FBTCS suffer from unfavorable treatment outcome compared to TLE patients without FBTCS is unclear. However, developing new treatment strategies for TLE-FBTCS requires a detailed understanding of the differences between the phenotypes and their underlying mechanisms.

Increased cerebral blood flow in temporal lobe in pre-generalization period, in medial cerebellum, thalamus and basal ganglia during generalization, and in bilateral cerebellar hemispheres, midbrain and basal ganglia post-ictally have been observed in patients with FBTCS [9]. A recent structural image study found that TLE patients with FBTCS showed atrophy of the pallidum, thalamus, and posterior cingulate cortex compared to a TLE-non-FBTCS group [8]. A microstructural image study of patients without FBTCS found the integrity of the ipsilateral hippocampal-thalamic pathway impaired, whereas patients with FBTCS showed bilateral hippocampal-thalamic pathway impairment. In addition, the patients with FBTCS exhibited greater impairment of the ipsilateral hippocampal-thalamic pathway than those without FBTCS [10]. He et al. [11] found that unilateral TLE patients without FBTCS had reduced thalamocortical functional connectivity (FC) in the ipsilateral epileptogenic focus, but patients with FBTCS presented reduced thalamocortical FC in both ipsilateral and contralateral epileptogenic foci. In addition to the thalamus and hippocampus, the basal ganglia also might take part in modulating generalized tonic-clonic seizures. Patients with FBTCS showed increased integration between the globus pallidus internus (GPi) and putamen, and decreased integration between GPi and thalamus. More and more evidence suggest that basal ganglia, thalamus, hippocampus and cerebellum might also play an important role in the propagation/control of TLE and modulation of FBTCS. Often studies of patients with TLE-FBTCS have only focused on imaging altered connectomics within specific brain regions [10–13]. However, surgery removing localized brain tissue often cannot completely solve the problem of epilepsy recurrence, suggesting that TLE is a more comprehensive brain network disease. The human brain connectome is recognized as a large-scale complex network and these studies indicate that it is necessary for us to study the properties of complex networks to better understand and treat TLE.

Graph theoretical analysis is a mathematical tool which provides a framework for quantifying and analyzing topological properties of a complex network. Previous network investigations of epilepsy have found that TLE and idiopathic generalized epilepsy with generalized tonic-clonic seizures (IGE-GTCS) exhibited altered global and nodal topological properties, i.e., TLE-FBTCS had a higher thalamic degree and betweenness centrality compared to TLE-non-FBTCS, which might relate to uncontrolled secondary generalization [14–18]. Though the cerebellum was shown to participate in propagation or control of seizures in previous studies, only a few graph-theoretical studies examining TLE-FBTCS patients have shed light on the importance of this structure [19, 20].

Previous studies have found that cognitive impairment is the most common comorbidity with epilepsy [21]. The forms of cognitive impairment in epilepsy vary widely, including disturbances in language, memory and executive control function, but attention deficits are particularly common [22]. Attention includes three components: alertness, orientation and executive control. Alertness is divided into two types: (1) intrinsic alertness, representing internal arousal or wakefulness without external stimuli, and (2) phasic alertness, representing the short-term ability to enhance response readiness subsequent to external stimuli [23]. Our previous studies found that patients with TLE demonstrated impaired intrinsic and phasic alertness performance, and these alertness deficits were negatively correlated with decreased thalamic FC with anterior cingulate cortex [24–26]. FBTCS is a strong predictor of cognitive decline, whereas few studies on the TLE-FBTCS have reported altered alertness [27, 28]. Another aim of our study was to explore whether there are differences in alertness between TLE patients with and without FBTCS.

In the current study, we hypothesized that there are different network properties between TLE patients with and without FBTCS. We chose an atlas containing the cerebellum and the cerebrum to construct a functional brain network based on resting state functional magnetic resonance imaging data for TLE patients with and without FBTCS and healthy controls respectively. This atlas was used to compare their network properties using the graph theoretical approach. We assessed the relationship between altered network properties and alertness or other clinical variables.

## Methods

### Participants

From January 2019 to March 2020, we recruited 39 consecutive patients with TLE, through the outpatient epilepsy clinic at the First Affiliated Hospital of Guangxi Medical University, Nanning, China. All patients had the typical clinical symptoms of temporal lobe seizures and were classified as TLE according to ILAE diagnosis and classification criteria [1, 29]. At the same time, all patients were diagnosed by 2 experienced epileptologists based on detailed clinical history, serial EEG studies (video-EEG was performed for suspected patients), neuroimaging results, and neuropsychological assessment. Seizures and interictal epileptiform discharges have been associated with cognitive dysfunction [30, 31]. Thus, all patients were taking antiepileptic drugs regularly and were free of seizures for least three months prior to participating in the study.

Patients were excluded for the following criteria: (1) history of cerebral organic diseases and mental diseases, such as brain trauma, brain surgery, brain tumor, stroke, multiple sclerosis, Alzheimer’s disease, schizophrenia, etc. (2) medical illness or infection with central nervous system impact other than epilepsy, (3) neuroimaging findings of abnormal changes in brain structures in MRI/CT images, except for hippocampal sclerosis, (4) ambiguous documentation of FBTCS history, or (5) unable to complete the whole experimental process.

All patients primarily presented with simple partial and/or focal complex seizures, and some had FBTCS, based on the theirs semiology and history of epilepsy [32]. TLE patients were assigned to the TLE-FBTCS group if they presented as FBTCS or had a history of FBTCS during their lifetime, or to the TLE-non-FBTCS group if they did not present as FBTCS. The patients were divided into FBTCS group (TLE-FBTCS) or without FBTCS group (TLE-non-FBTCS) [8, 11]. Since the lateralization of seizure focus is considered to be a confounding factor associated with changes of topological properties of brain networks in patients with TLE [33]. TLE patients with and without FBTCS were further divided into four subgroups: (1) LTLE-FBTCS: left TLE with FBTCS (n = 10), (2) LTLE-non-FBTCS: left TLE without FBTCS (n = 7), (3) RTLE-FBTCS: right TLE with FBTCS (n = 11), and (4) RTLE-non-FBTCS: right TLE without FBTCS (n = 11).

The typical clinical manifestations of FBTCS are bilateral limb rigidity and secondary rhythmic clonic jerks. These symptoms are characterized by bilateral asymmetry and asynchrony [11, 32, 34]. The classification of seizures was based on the clinical description of the witnesses and seizure videos. Twenty-two demographically matched healthy controls (HCs), with no history of psychiatric or central nervous system diseases and no evidence of structural abnormalities on intracranial MRI/CT images, were recruited. The HC group was further divided into two subgroups balanced for age and gender (HC-1 and HC-2). The details of demographic and clinical measures of the group and subgroups are summarized in Tables 1, 2 and 3. We applied the Edinburgh Hand Preference Inventory to identify handedness [35]. The Medical Research Ethics Committee of the First Affiliated Hospital of Guangxi Medical University approved this study [No. 伦审(2018-KY-国基-040)] and informed consent was collected from all participants before the study. The trial was performed in accordance with the Declaration of Helsinki.

**Table 1:** The demographic, clinical characteristic and alertness of two groups with TLE and HCs

		TLE-non-FBTCS (18)	TLE- FBTCS (21)	Health control (22)	P value
Gender (M/F)		8/10	11/10	9/13	0.744(Chi-square)
Age (M ± SD)		29.722 ± 8.857	33.523 ± 8.376	29.772 ± 6.279	0.212(ANOVA)
Education years, median (range)		15(9-16)	12(9-16)	15(10-18)	0.103(Kruskal-Wallis)
Side (R/L)		11/7	11/10	/	0.584(Chi-square)
Hippocampal sclerosis (Yes or No)		8/10	12/9	/	0.429(Chi-square)
Age of epilepsy onset (M ± SD)		21.278 ± 10.665	21.238 ± 11.144	/	0.991(T-test)
Number of seizure years, median (range)		5.5(1-21)	11.0(3-35)	/	0.068 (Mann-Whitney)
Type of seizure	FAS	5	/	/	/
	FIAS	10	/	/	/
	FAS + FIAS	3	/	/	/
	FAS + FBTCS	/	2	/	/
	FIAS+FBTCS	/	19	/	/
Alertness	Alertness effect (M ± SD)	0.106 ± 0.037	0.078 ± 0.034	0.090 ± 0.033	0.043(ANOVA)
	No cue RT (M ± SD) ms	669.49 ± 87.15	715.87 ± 129.06	606.29 ± 72.22	0.003(Kruskal-Wallis)
	Double cue RT median (range)ms	616.32 (455.52-746.40)	701.68 (569.89-1085.79)	547.76 (457.38-708.38)	0.003(Kruskal-Wallis)
ASM (mono or polytherapy)		5/13	7/14	/	0.708(Chi-square)

*HCs* Health controls, *M* Man, *F* Female, *M ± SD* Mean ± standard deviation, *ASM* Anti-seizure medications, *FAS* Focal aware seizures, *FIAS* Focal impaired awareness seizures, *FBTCS* Focal to bilateral tonic–clonic seizures, *ANOVA* One-way analysis of variance, *χ2* Chi-square tests; NA

**Table 2:** The demographic, clinical characteristic and alertness of the two subgroups with right TLE and HCs-1

		RTLE-non-FBTCS (11)	RTLE- FBTCS (11)	HCs-1 (11)	P value
Gender (M/F)		5/6	6/5	5/6	0.886(Chi-square)
Age (M ± SD)		29.091 ± 8.240	34.818 ± 8.208	28.909 ± 6.379	0.138(ANOVA)
Education years,median (range)		15(9-16)	15(9-16)	16(12-17)	0.059(Kruskal-Wallis)
Hippocampal sclerosis (Yes or No)		7/4	6/5	/	1.00
Age of epilepsy onset (M ± SD)		20.909 ± 9.721	22.818 ± 9.527	/	0.647(T-test)
Number of seizure years,median (range)		5(1-21)	13(6-20)	/	0.080 (Mann-Whitney)
Type of seizure	FAS	2	/	/	/
	FIAS	6	/	/	/
	FAS + FIAS	3	/	/	/
	FAS + FBTCS	/	1	/	/
	FIAS+FBTCS	/	10	/	/
Alertness	Alertness effect (M ± SD)	0.099 ± 0.034	0.070 ± 0.031	0.085 ± 0.034	0.126(ANOVA)
	No cue RT (M ± SD)	688.49 ± 85.32	725.23 ± 165.39	670.69 ± 125.88	0.047(ANOVA)
	Double cue RT median (range)	655.17 (455.52-746.40)	632.94 (529.01-1082.13)	518.72 (486.47-708.38)	0.041(Kruskal-Wallis)
AEDs (mono or polytherapy)		3/8	2/9	/	0.611(Chi-square)

*HCs* Health controls, *M* Man, *F* Female, *M ± SD* Mean ± standard deviation, *ASM* Anti-seizure medications, *FAS* Focal aware seizures, *FIAS* Focal impaired awareness seizures, *FBTCS* Focal to bilateral tonic–clonic seizures, *ANOVA* One-way analysis of variance, *χ2* Chi-square tests; NA

**Table 3:** The demographic, clinical characteristic and alertness of the two subgroups with left TLE and HCs-2

		LTLE-non-FBTCS (7)	LTLE-FBTCS (10)	HCs-2 (11)	P value
Gender (M/F)		3/4	5/5	4/7	0.663(Chi-square)
Age (M ± SD)		30.714 ± 10.355	32.100 ± 8.761	27.091 ± 5.991	0.372(ANOVA)
Education years,median (range)		15(9-16)	14(9-16)	13(10-18)	0.507(Kruskal-Wallis)
Hippocampal sclerosis (Yes or No)		1/6	6/4	/	0.166
Age of epilepsy onset (M ± SD)		21.714 ± 13.499	21.600 ± 8.487	/	0.983(T-test)
Number of seizure years, median (range)		9(3-22)	6.000(2.0-22)	/	0.922(Mann-Whitney)
Type of seizure	FAS	3	/	/	/
	FIAS	4	/	/	/
	FAS + FIAS	/	/	/	/
	FAS + FBTCS	/	1	/	/
	FIAS+FBTCS	/	9	/	/
Alertness	Alertness effect (M ± SD)	0.117 ± 0.042	0.086 ± 0.038	0.086 ± 0.033	0.182(ANOVA)
	No cue RT (M ± SD)	624.87 ± 75.01	705.56 ± 79.96	604.02 ± 59.12	0.009(ANOVA)
	Double cue RT (M ± SD)	572.95 ± 74.81	651.04 ± 81.62	554.58 ± 59.84	0.014(ANOVA)
ASM (mono or polytherapy)		3/4	5/5	/	0.772(Chi-square)

*HCs* Health controls, *M* Man, *F* Female, *M ± SD* Mean ± standard deviation, *ASM* Anti-seizure medications, *FAS* Focal aware seizures, *FIAS* Focal impaired awareness seizures, *FBTCS* Focal to bilateral tonic–clonic seizures, *ANOVA* One-way analysis of variance, *χ2* Chi-square tests; NA

### Neuropsychological assessment

All subjects were evaluated for alertness with a shortened version of the Attention Network Test (ANT, the version of AttentionExp1.1B5), which was a combination of Flanker task and cued RT task [36]. The method used to evaluate alertness in the present study is the same as in our previous study [24, 37, 38]. The version applied to the current study consisted of three blocks, each containing 96 trials. During evaluation of alertness, participants sat 60 cm from the computer and were required to gaze at the cross on center of the screen. In each trial, the subject pressed as quickly and precisely as possible on the mouse button corresponding to the direction of the target arrow that appeared on the screen. There could be three different, randomly appearing combinations for the directions of the target arrow and the flanker, different directions (incongruent), uniform directions (congruent), or without any direction (neutral). Before the target arrow appeared, an asterisk served as one of four possible warning cues: center cue, spatial cue, double cue, or no cue. The E-prime software (Psychology Software Tools, Pittsburgh, PA) recorded both correct and incorrect reaction times (RT). We only analyzed the correct trials and filtered out all RT > 1500 ms and < 200 ms. To eliminate the influence of age, we calculated the alertness effect using the following formula: $\mathrm{Alertness}=\left({\mathrm{RT}}_{\mathrm{no}\ \mathrm{cue}}-{\mathrm{RT}}_{\mathrm{double}\ \mathrm{cue}}\right)\left/ {\mathrm{RT}}_{\mathrm{double}\ \mathrm{cue}}\right.$. The ratio effect scores were widely used in recent studies of alertness functions [37, 39].

### Image acquisition

All image data were obtained on a 3.0 T MRI system (Philips, The Netherlands) with a 12-channel phase array head coil at the First Affiliated Hospital of Guangxi Medical University. The image acquisition parameters were as follows: repetition time/echo time (TR/ TE) =2000/30 ms, matrix size =64 × 64, flip angle = 90°; slice thickness = 3.5 mm; slice gap = 0.5 mm; field of view = 220 mm × 220 mm, and voxel size = 3.44 mm × 3.44 mm × 4 mm. A total of 225 volumes (41 slices per volume) were collected from all participants with a gradient-echo planar image sequence acquired over 450 s. All participants laid on a pad to comfortably fix the head and wore a headset to reduce noise. They were instructed to stay still, keep their eyes closed, stay awake, and avoid thinking about anything in particular, during the entire MRI scanning procedure. MRI scanning began 15-30 min after they finished the ANT, between 5 pm to 8 pm.

### Image date preprocessing

All functional MRI datasets were preprocessed and analyzed using SPM12 (http://www.fil.ion.ucl.ac.uk/spm/software/spm12/) and the GRETNA software (http://www.nitrc.org/projects/gretna/) [40]. For each individual dataset, the first 10 volumes were removed to ensure signal equilibration. The remaining 215 volumes were corrected for slice-timing and head movement, normalized to the Montreal Neurological Institute (MNI) stand space using the Echo Planar Imaging (EPI) template, and resampled to 3 mm × 3 mm × 3 mm isotropic voxels. Subsequently, the images were smoothed using a 6 mm full width at half maximum Gaussian kernel and regressed to minimize confounding signals (Friston-24 head motion parameters, the averaged white matter signal, and the cerebrospinal fluid signal, and bandpass filtered (0.01-0.08 Hz). All data met the criterion of translation <2 mm and rotation <2°.

### Function network construction and analysis

We used GRETNA v 2.0.0 software (http://www.nitrc.org/projects/gretna/) to construct the topological brain networks for each individual and analyze the graph characteristics of functional connectivity networks. After preprocessing the image data, the function images were segmented into 160 anatomical regions following the Dosenbach 160 atlas [41]. In a functional network, each cerebral region represented a node. Representative mean time series for each region were acquired by averaging the time series across all its voxels. The values of Pearson correlation coefficients between the mean time series for each node served as the edges. After eliminating negative correlations, we obtained a binary unweighted graph. We used GRETNA software to pre-calculate the topological properties of all our subjects and found that graph topology becomes small-world (small-worldness >1) in the sparsity range 17-50% with steps of 0.01. Below a connection density of 17%, some graphs began to fragment, and above a connection density of 50%, graph topology became increasingly random and less small-world [42, 43].

At each sparsity threshold, we calculated global and nodal network measurements for all subjects. To mitigate the issue of multiple comparisons across network sparsity ranges and increase our sensitivity, we computed the area under the curve statistic (AUC) across sparsity ranges for each topological measure using GRETNA software. For each topologic measurement, we calculated the AUC within the sparsity range. The AUC values of the global and nodal measurements were compared across groups or subgroups. The global measurements included small-worldness (σ), normalized clustering coefficient (γ), normalized characteristic path length (λ), global network efficiency (gE) and local network efficiency (locE). $\mathrm{small}-\mathrm{worldness}=\mathrm{normalized}\ \mathrm{clustering}\ \mathrm{coefficient}\left/ \mathrm{normalized}\ \mathrm{characteristic}\ \mathrm{path}\ \mathrm{length}\right.$ Small-world networks have relatively high clustering and path length, with an intermediate topological organization compared to random and regular networks [37, 42]. The normalized clustering coefficient (γ) equals the ratio of clustering coefficients in real and random networks and the normalized characteristic path length (λ) refers to the ratio of characteristic path lengths in real and random networks. The clustering coefficient refers to the number of connections that exist between a node and its nearest neighbors, and the characteristic path length is the average of the shortest path between two nodes. The global network efficiency (gE) is the reciprocal of the mean shortest path in the network, which measures the ability of information transmission between network nodes, and the shortest path is the minimum distance between edges of any pair of nodes. The local efficiency is the mean reciprocal of the shortest paths between one node and its neighboring nodes, which reflects the fault tolerance and how efficient the mutual communication is among sub-networks composed of neighboring nodes [16, 44, 45].

Five local metrics were applied to describe the properties of nodes. These included: (1) the degree of centrality (DC), (2) betweenness centrality (BC), (3) nodal efficiency (NE), (4) nodal local efficiency (NLe), and (5) nodal shortest path (NLp).

The global and local parameters were initially compared between the TLE-non-FBTCS, TLE-FBTCS, and HC groups. Subsequently these were compared between the subgroups of RTLE-FBTCS, RTLE-non-FBTCS and HC-1. Finally, these parameters were compared between the subgroups of LTLE-FBTCS, LTLE-non-FBTCS and HC-2.

### Statistical analysis

Data analysis was performed using SPSS (v16; IBM) and GRETNA software packages. Chi-square tests were used to compare categorical variables (e.g., gender, side and AEDs). The clinical characteristics were compared between the patients with and without FBTCS using Mann-Whitney U test or two independent samples t-test in the SPSS (v16; IBM). To identify differences in demographic and alertness factors among TLE-non-FBTCS, TLE-FBTCS and HC groups, one-way analysis of variance (ANOVA) (P < 0.05) was performed. Kruskal-Wallis one-way ANOVA of ranks was used to compare data of variables not normally distributed and variance was not heterogeneous. When significant differences (P < 0.05) were found among the three groups, Scheffe’s test was used for post hoc analysis. For analyzing the characteristics of a network, we conducted a one-way ANOVA (P < 0.05) among the three groups using age and gender as covariates, and False discovery rate (FDR) correction was applied for multiple comparisons in the GRETNA software. In the subsequent pairwise comparisons, we first conducted two sample t-test in GRETNA using FDR correction (P < 0.05) and then Bonferroni correction as a second correction for multiple comparisons [37]. All t-tests were two-tailed, with a significance level of P < 0.05. To investigate the relationships between network metrics and the number of seizure years or alertness measures of TLE patients with or without FBTCS, we analyzed significantly altered topological properties and the number of seizure years or alertness scores (intrinsic alertness, phasic alertness and alertness effect). Pearson’s correlation analysis was applied to data with a normal distribution, and Spearman’s correlation analysis was applied to data with a non-normal distribution. Bonferroni corrections were used for multiple comparisons. The threshold of corrected p-value was less than 0.05/16 = 0.0031. The relationship of the altered topological property with alertness measures or number of seizure years were evaluated in SPSS (v16; IBM). We performed correlation analyses between altered topological properties and behavioral measures or number of seizure years in the whole study sample, i.e., initially by pooling all study subjects together independent of group allocation, and then separately by partitioning the data into the three groups. The same statistical methods were applied to compare the subgroups of right TLE patients to HC and left TLE patients to HC.

### Visualization of results

We applied for BrainNet Viewer to show the results of the group nodal topological characteristics [46].

## Results

### Demographic, clinical characteristic and alertness

There were no significant differences in age, education years and gender levels between the TLE-FBTCS, TLE-non-FBTCS and HC groups. Also, no statistically significant differences were observed in the age of onset, the number of seizure years, hippocampal sclerosis and lateralization of the epileptogenic zone between the groups of TLE-FBTCS and TLE-non-FBTCS (Table 1). The two subgroups of right or left TLE patients and the subgroup of healthy controls were statistically similar in age, education years and gender. The two subgroups of right TLE patients also did not differ in clinical characteristics. Similarly, no differences were detected between the two subgroups of left TLE and HC-2 (Tables 2 and 3).

**Fig. 1:**
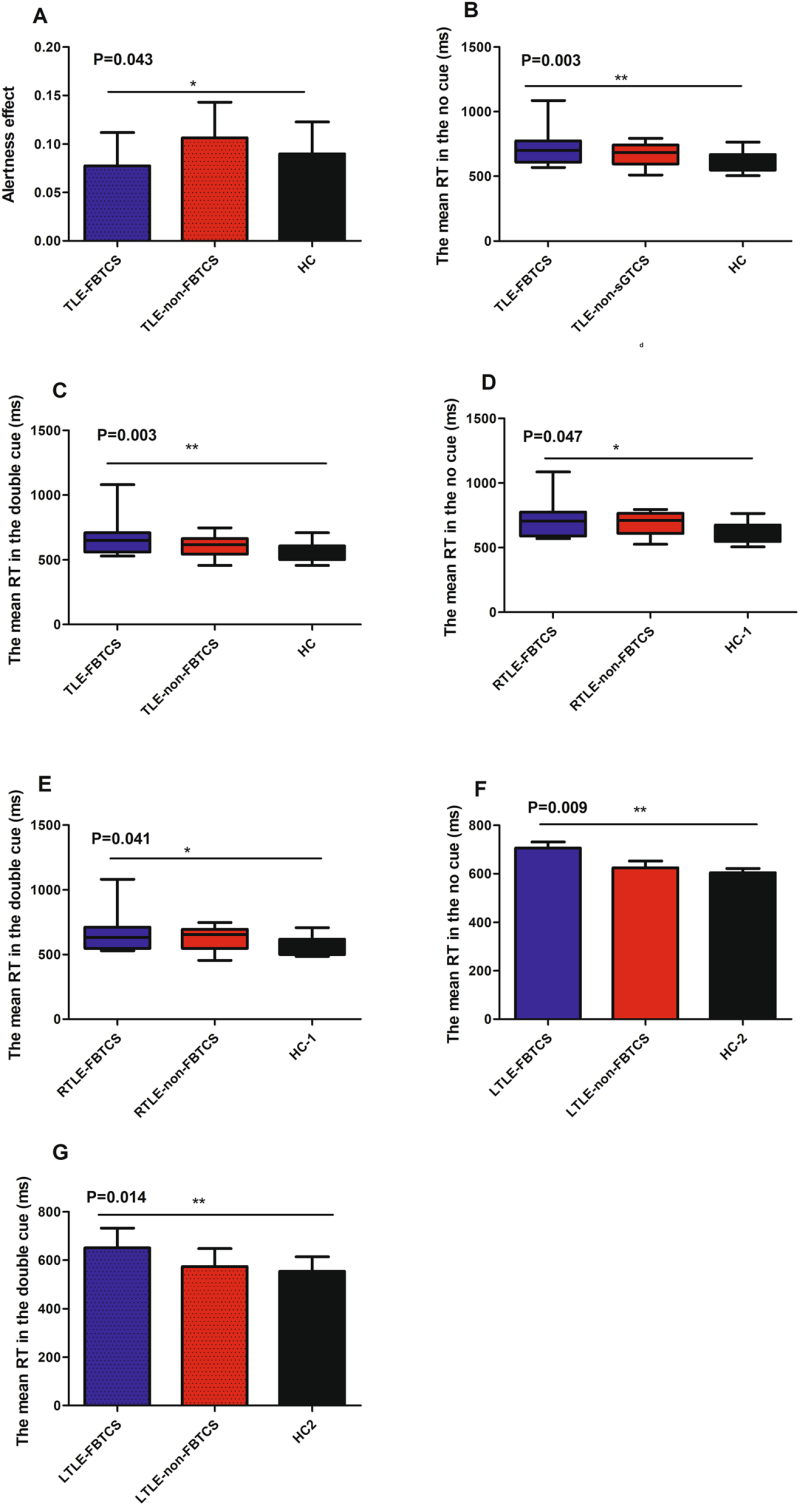
Comparisons of Neuropsychological results for the patients with TLE and HC. P-values represent significant ANOVA F-ratios. The significance of post hoc comparisons is denoted by *. **A** The results of alertness effect in two patient groups (TLE-non-FBTCS and TLE-FBTCS) and HC. **B** and **C** The results of the mean RT in no cue and double cue trials in two patient groups (TLE-non-FBTCS and TLE-FBTCS) and HC, respectively. **D** and **E** Same as **B** and **C**, but for the patients with right TLE and HC-1. **F** and **G** Same as **B** and **C**, but for the patients with left TLE and HC-1. ***P < 0.001, **0.001 < P < 0.01, * 0.01 < P < 0.05

The TLE-FBTCS patients exhibited the longest mean RT in the no cue and double cue trials, and the lowest ratio effect scores of all three groups (Fig. 1 A, B and C). The subgroups of RTLE-FBTCS and LTLE-FBTCS showed similar mean RT results in no cue and double cue trials (Fig. 1 D, E, F and G). All subjects were right-handed in this study.

### Altered global network measurements in patients

**Fig. 2:**
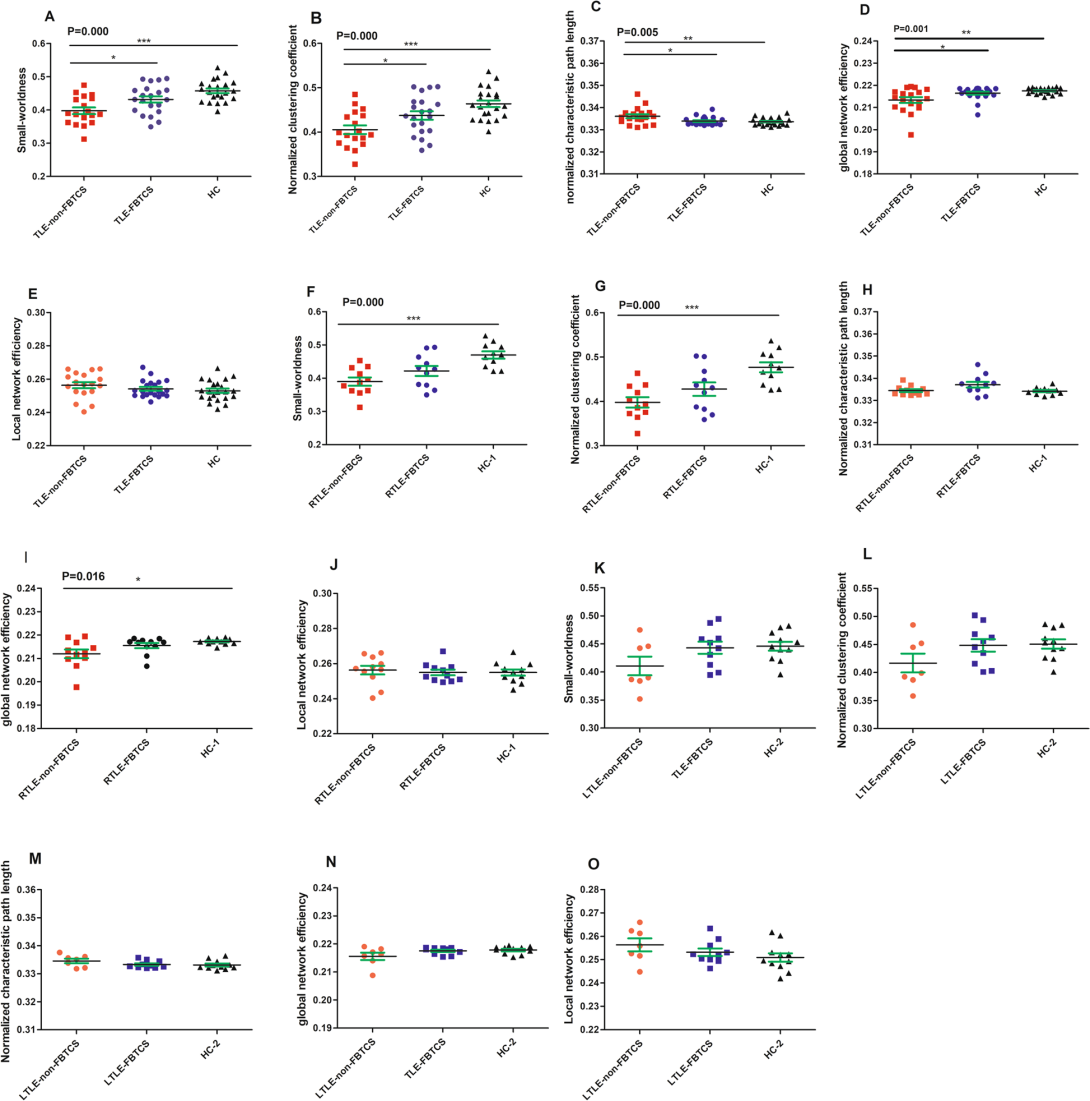
The global characteristics of patients without FBTCS were significantly altered. P-values represent significant ANOVA F-tests. The significance of pairwise comparison t-test with Bonferroni-Holm correction for multiple comparisons are denoted by *. **A**, **B**, **C**, **D** and **E** show small-worldness, normalized clustering coefficient, normalized characteristic path length, global network efficiency and local network efficiency in two patient groups (TLE-non-FBTCS and TLE-FBTCS) and HC, respectively. **F**, **G**, **H**, **I** and **J** are the same as **A**, **B**, **C**, **D** and **E** but for the patients with right TLE and HC-1, respectively. And **K**, **L**, **M**, **N** and **O** are the same as** (A), (B), (C), (D**) and **E** but for the patients with Left TLE and HC-2, respectively. *** P < 0.001, ** 0.001 < P < 0.01, * 0.01 < P < 0.05

At global measurement levels, small-worldness and global network efficiency were reduced in two groups of patients compared with HCs (Supplementary table). We found that small-worldness, normalized clustering coefficient and global network efficiency scores of the TLE-FBTCS group exhibited degression from the HC group and were lowest in the TLE-non-FBTCS group (Fig. 2 A, B and D). The normalized characteristic path lengths were higher in the group with FBTCS than HC subjects and were highest in the group without FBTCS (Fig. 2 C). Among the three groups, the TLE-non-FBTCS group had significantly lower values in small-worldness, normalized clustering coefficient, and global network efficiency. The patients with TLE-FBTCS showed significantly higher small-worldness, normalized clustering coefficient and global network efficiency, and lower normalized characteristic path lengths than the patients with TLE-non-FBTCS. However, the last comparison did not pass the Bonferroni correction for multiple comparisons, and there were no significant differences between three groups in any other global measurements (Fig. 2 A, B, C, D and E). We also observed a similar change in small-worldness, and normalized clustering coefficient between the two subgroups right TLE and HC-1 subgroup (Fig. 2 F, G, H, I and J). However, no significant alterations of global measures were observed between the subgroups of left TLE and HC-2 subgroups (Fig. 2 K, L, M, N and O).

### Altered nodal efficiency in patients

**Fig. 3:**
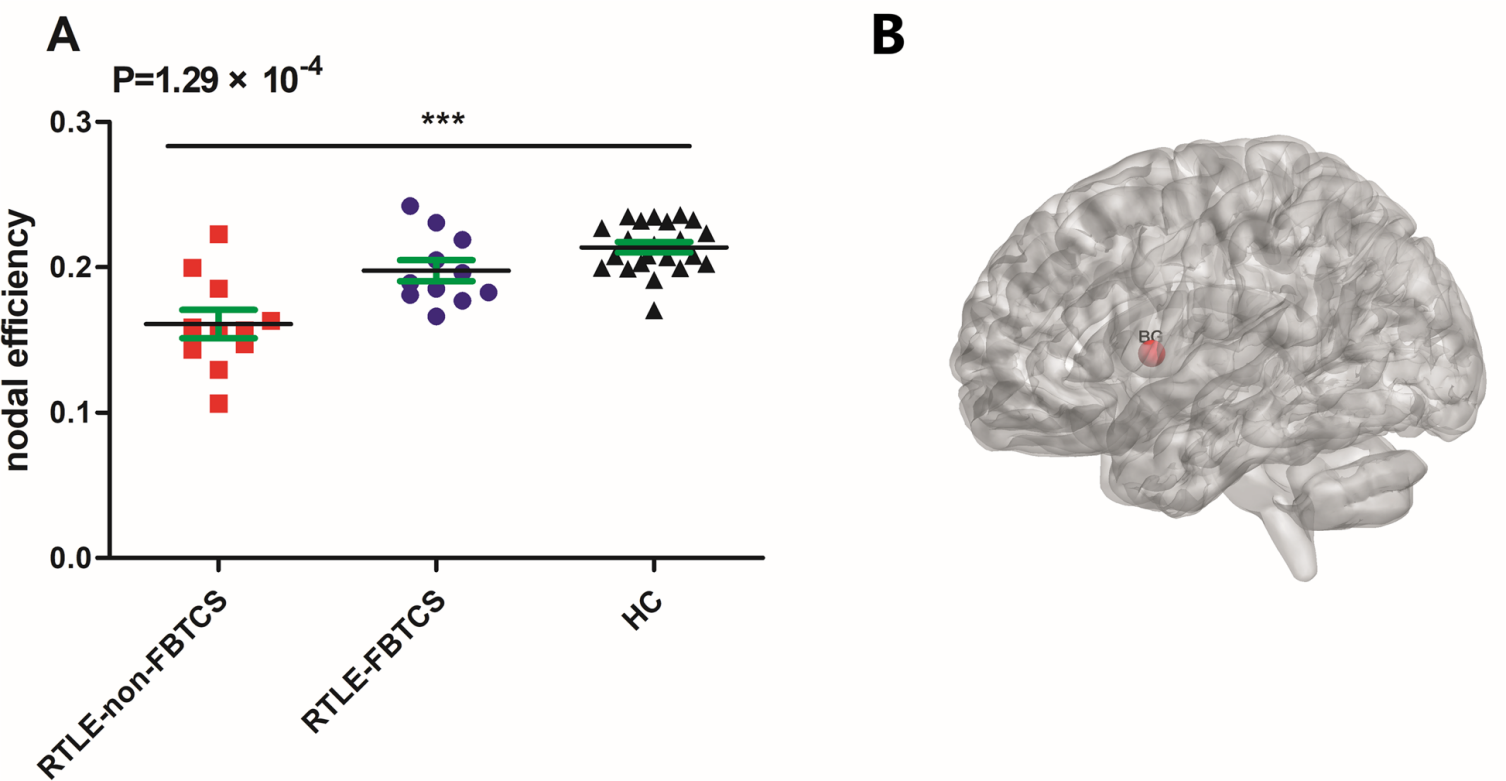
The nodal characteristic is diminished for the patients with right TLE-non-FBTCS. P-values represent significant ANOVA F-tests. **A** nodal efficiency in two patient groups (right TLE-non-FBTCS and right TLE-FBTCS) and HC-1. The significant alteration did not survive analysis by Bonferroni correction for pairwise comparison. **B** Location of the basal ganglia

In addition to global measurement level comparisons between groups, we also investigated which nodes demonstrated a significant change in nodal measurement levels compared to HC groups. No significant alterations of nodal measurements were presented between the groups with TLE-non-FBTCS, TLE-FBTCS and HC. However, we only found that the nodal efficiency significantly decreased in basal ganglia contralateral to the epileptogenic focus of the subgroup with RTLE-FBTCS compared to HC-1 and were lowest in the subgroup with RTLE-non-FBTCS. The subgroup with RTLE-non-FBTCS showed a trend of lower nodal efficiency scores for the contralateral basal ganglia than those of the HC. However, this did not survive the Bonferroni correction for pairwise comparison (Fig. 3 A and B). There were no significant alterations of nodal measurements observed between the two subgroups of LTLE and HC-2.

### Relationships between topological properties and number of seizure years or alertness

**Fig. 4:**
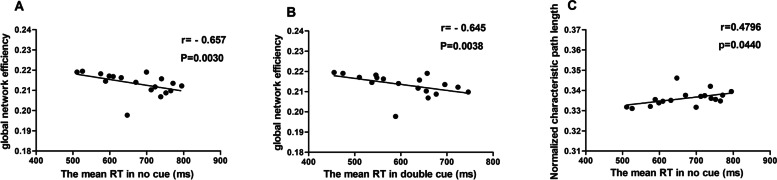
Correlations of significantly altered topological properties and alertness in patients without FBTCS. **A** The correlation of global network efficiency and mean RT in double cue trials of patients without FBTCS. **B** The correlation of global network efficiency and mean RT in double cue trials of patients without FBTCS. **C** The correlation of characteristic path length and mean RT in no cue trials of patients without FBTCS. The threshold of corrected p-value was less than 0.0031 and r represented the correlation coefficient

We found that global network efficiency was negatively correlated with intrinsic alertness in the group of TLE-non-FBTCS, a significant correlation which survived multiple comparisons corrections (Fig. 4 A). We also detected a significant negative correlation between phasic alertness and global network efficiency, and a significant positive correlation between intrinsic alertness scores and characteristic path length in the patients with TLE-non-FBTCS (Fig. 4 B and C). However, neither correlation was significant after Bonferroni correction for multiple comparisons. There were no significant correlations between altered topological properties and duration of seizure years or behavioral measures in the whole study sample. Also, there were no significant correlations between altered topological properties and duration of seizure years or alertness (intrinsic alertness, phasic alertness and alertness effect) among two subgroups of RTLE and HC-1, or between the two subgroups of LTLE and HC-2.

## Discussion

In the current investigation, the functional brain networks of the groups with TLE-FBTCS, TLE-non-FBTCS and HC were constructed respectively, based on resting-state fMRI. To differentiate the topological characters of the three groups, we utilized graph theoretical analysis to compare the topological properties of functional brain networks. The four main results of our study are as follows: (1) Compared with TLE-non-FBTCS, TLE-FBTCS showed significant alterations of small-worldness γ, and a trend of significant alterations of gE (i.e. a significant difference of gE was observed between TLE-non-FBTCS and TLE-FBTCS, but no significant deviations between RTLE with and without FBTCS), indicating there might be different disorders in brain network information integration and separation between them; (2) Compared with the HC subgroup, the subgroup of right TLE without FBTCS had altered nodal efficiency in the contralateral basal ganglia, disrupting the role of these nodes in the process of information transmission and integration; (3) The patients with TLE-FBTCS had poorer performance than those with TLE-non-FBTCS in relation to intrinsic/phasic alertness and alertness effects, suggesting that FBTCS might predispose a patient with TLE to deteriorate in alertness; (4) In the present study, we observed that gE might exhibit a significant negative correlation with intrinsic alertness in patients with TIE-non- FBTCS.

### Altered global topology of functional network

In our study, the results revealed that the brain functional networks for TLE-FBTCS, TLE-non-FBTCS, and HC showed a prominent small-world topological characteristic which was consistent with previous graph theoretical investigations on epilepsy [16, 17, 37]. Such a small-world network is considered to be the most optimized network organization related to transinformation’s segregation and integration processing [14]. However, the TLE patients with and without FBTCS in this study showed aberrant small-worldness, suggesting they might have a less efficient network topology relevant to their pathological state compared to HCs.

Γ plays a role in measuring the strength of the network segregation function and is an important indicator for checking whether the small-worldness network becomes more regular or random. λ quantifies the integrated function of network information, and is used together with γ to distinguish between three types of networks: regular, random, and small-world networks. The regular network exhibits large γ, long λ, higher local segregation, and low global integration ability, whereas the random network shows small γ, short λ, lower local segregation, and higher integration function. The small-world network has large γ and short λ, with the best integration and separation functions [14, 41, 44, 47]. gE is defined as the reciprocal of the mean length of the shortest path between nodes in a network, and is a measure of the ability for information exchange between these nodes. A lower gE reflects a longer distance between nodes and slower long-range communication within the networks [16, 44]. The group of TLE-non-FBTCS patients showed significantly lower small-worldness, γ and gE, and longer λ than HC, findings consistent with some previous investigations applying graph theory approaches to study brain networks of epilepsy. These findings suggest an impairment in segregation, integration function and slower long-distance information communication for the TLE-non-FBTCS [16, 33, 47]. The patients with TLE-FBTCS demonstrated significantly higher small-worldness, γ and gE, a trend of decreasing in λ compared to the group of TLE-non-FBTCS, and a trend of decreasing small-worldness, γ and gE, and increasing λ compared to HC. These results identify differences in segregation function, integration function, and capability of information communication between TLE-FBTCS and TLE-non-FBTCS patients. The brain network of TLE-FBTCS exhibited a trend of decreasing small-world and γ characteristics compared to HC, similar to a previous graph theoretical study on idiopathic generalized epilepsy with generalized tonic-clonic seizure [17]. The results indicated that the brain network of TLE-FBTCS also might have lower segregation function than healthy controls. Moreover, the comparison of the topological characteristics of the two subgroups with RTLE and HC-1 showed the subgroup of RTLE-non-FBTCS was similar to the group of TLE-non-FBTCS patients, and the subgroup of RTLE-FBTCS patients demonstrated a trend of altered global measurement which was analogous to TLE-FBTCS patients, further confirming the above conclusion. Recently, Sinha et al. reported that TLE-FBTCS showed a greater and more widespread extent of abnormal structural network whereas TLE-non-FBTCS exhibited more localized changes, results that seem to be inconsistent with ours [48]. Why do TLE-FBTCS patients seem to have near normal topological measurements compared to TLE-non-FBTCS patients in the present study? One reason might be the neuroplasticity of the human brain. Seizure-inducted plasticity may have “bidirectional” properties, which induces progressive cumulative injury while also inducing resistance to additional damage, which may be an effect of timing of seizures or inter-seizure interval [49]. Compared with patients with a shorter course of epilepsy, the brain with a longer course of epilepsy has more time to modulate structural or functional connectivity to compensate for this pathological state. Our previous research found that this process was gradual and may take 5 years or more [50]. In the present study, compared with the non-FBTCS group, FBTCS patients seem to have a longer course of epilepsy, which means that the functional connectivity of FBTCS patients might be closer to normal level than non-FBTCS patients. Another reason may be that the small sample of patients was insufficient to determine if the abnormal topological properties of TLE-FBTCS patients are more widespread than those of TLE-non-FBTCS patients. We constructed functional brain networks for the TLE patients with and without FBTCS, respectively, compared their topological properties, and found that there were significantly different small-worldness and γ, and a trend of significant difference of gE between the groups. We speculate that the patients with TLE-FBTCS might have a global topological organization related to the pathological state that is different from the patients with TLE-non-FBTCS.

### Altered nodal efficiency in RTLE-non-FBTCS patients

Nodal efficiency is defined as the average of the derivative of the shortest path length between one node and the anther node, and quantifies the parallel information transmission capacity of one node in the network. The higher the node efficiency, the stronger the communication ability [51, 52]. When we compared the characteristics of nodal network of RTLE patients and HC, we found that the subgroup of RTLE-non-FBTCS had a trend of lower nodal efficiency in the basal ganglia of the left cerebral hemisphere than the subgroups of RTLE-FBTCS and HC-1. However, there were no significant differences of topological measurements found in patients with left TLE and HC-2. The reason might be the distinctive patterns of FC between right and left TLE, which appears more seriously disrupted in right TLE, [33, 53] or stem from a difference in patients’ semiology. The nodal efficiency in the basal ganglia of the left cerebral hemisphere showed a tendency for more efficiency in the subgroup of RTLE-FBTCS than the subgroup RTLE-non-FBTCS, and efficiency was highest in HC-1. This suggests that the left basal ganglia in the RTLE subgroup might have a lower communication ability compared to HC. The basal ganglia, a group of interconnected subcortical nuclei, are involved in motor control, cognition, and motivational behaviors. The basal ganglia receive projections from neocortex, thalamus, hippocampus and amygdala. Conversely, they have output projections to the thalamus and the brainstem [54]. The basal ganglia play the role of a “braking system” between the cortex and the thalamus, regulating their activity by “direct”, “indirect” and “hyperdirect” pathways. TLE is a common focal epilepsy originating from temporal lobe structures, such as hippocampus, amygdala, entorhinal cortex, piriform cortex and temporal neocortex. The thalamus, hippocampus and amygdala participate in the process of generation, propagation and termination of seizures. The cortico-basal ganglia-thalamic loop plays an important part in the propagation or control of several types of seizures. Some evidence suggests that the basal ganglia play an inhibitory role in the propagation or control of TLE, but how this inhibition affects propagation and control of seizures are not yet clearly elucidated [55]. When the basal ganglia-thalamocortical loop is disrupted, the balance between basal ganglia inhibition and thalamic synchronization is broken, which may lead to focal to secondarily generalized tonic-clonic seizures [13]. The cortico-basal ganglia-thalamic circuit is not only involved in FBTCS, but the basal ganglia-cortical connectivity ipsilateral to the epileptogenic zone have been found to be reduced in focal epilepsy [56]. In previous investigations on perfusion changes with TLE using SPECT, some studies have shown decreases in [^18^F]fluoro-L-dopa uptake in the ipsilateral basal ganglia [57], but Bouilleret et al. [58] found a bilateral reduction in [^18^F]fluoro-L-dopa uptake in the basal ganglia in patients with TLE. In the present study, we found altered nodal efficiency in basal ganglia contralateral to the epileptogenic focus. The probable reason for this discrepancy might be related to the effects of AEDs on topological properties, heterogeneous seizure types in patients with RTLE-non-FBTCS, and the severity of the epileptic seizures [58, 59].

The left basal ganglia belong to a cingulo-opercular network (CON) [60]. CON is primarily associated with a wide range of cognitive processes, on-going performance monitoring and set-maintenance across multiple trials of a task. Disruption of this network connectivity may lead to abnormal cognitive control, emotional regulation and executive functioning. The associated epilepsy exhibits reduced FC in the cingulo-opercular network compared to controls [61, 62].

In the current study, we found that the RTLE-non-FBTCS patients exhibited a tendency of longer mean RT in no cue and double cue trials than those of HC. Combined with previous studies, we speculate that both of intrinsic alertness and phasic alertness might be impaired in the patients with RTLE-non-FBTCS [20, 24, 25].

### Alertness relevance of topological properties alterations

The patients with TLE-FBTCS exhibited significantly longer mean RT in no cue and double cue trials, and a lower ratio of scale than HC subjects, suggesting TLE-FBTCS patients have impaired intrinsic alertness, phasic alertness and alertness effect. Similarly, the patients with TLE-FBTCS showed a tendency of longer mean RTs in no cue and double cue trials and a lower ratio of scale compared to the patients with TLE-non-FBTCS. This suggests that the patients with TLE-FBTCS might have poorer performance than those with TLE-non-FBTCS in relation to intrinsic alertness, phasic alertness and alertness effect. These results are congruent with previous studies [15, 20]. One study reported that FBTCS was associated with cognitive decline [27]. We inferred that FBTCS might exacerbate the impairment of alertness in patients with TLE. However, we did not observe any correlation between altered topological properties and alertness in patients with TLE-FBTCS. The limited number of patients with TLE-FBTCS and the effects of heterogeneous histories of FBTCS patients on FC might be reasons for the disappointing results.

However, we found that gE was negatively correlated with mean RTs in no cue trials for the group of TLE-non-FBTCS patients. gE quantifies the network’s ability for parallel information dissemination between one node and another by multiple parallel pathways, and is related to integrated processing for cognitive functions. The more serious the deficit is in intrinsic alertness, the lower the gE and the poorer brain capability for integrating information. Some papers have reported that alertness is positively correlated with regional efficiency of the left thalamus, right inferior parietal gyrus and pallidum, and negatively correlated with nodal efficiency in the left middle temporal gyrus for HCs [63, 64]. Liang et al. recently reported a negative relationship between gE and alerting components in TLE patients with short duration (<5 years) [50]. In the current study, we also found that gE was negatively correlated with intrinsic alertness in the group of RTLE-non-FBTCS patients. In a previous study, our team did not find a relationship between alertness performance and gE in RTLE patients [15]. We also did not find any relationship between gE and alerted components in subgroup of RTLE-FBTCS in present study. We speculate that the decreased gE in RTLE-non-FBTCS patients might act as plasticity in response to disrupted intrinsic alertness. However, no similar relationship between gE and intrinsic alertness was observed in TLE-FBTCS, which may suggest excessive plasticity adaptive to the disrupted alertness performance in these patients. Although we did not find any correlational relationship between gE and alerted components in subgroup of LTLE with and without FBTCS, the limited samples may cancel out possible correlational relationships between gE and alerted components in the LTLE subgroups influenced by seizure lateralization.

### Limitations

We admit that there are several limitations in our study. First, the sample sizes were too small to detect the prominent difference in properties of networks among the three groups and subgroups. Our results should be reproduced by a future study with larger sample sizes. Second, we did not consider how different histories with respect to FBTCS might affect brain FC because of limited sample sizes. Although they had comparable number of seizure years, the patients diagnosed with FBTCS within the past year might have more serious disruptions of brain FC compared with patients with more remote histories of FBTCS (none for >1 year) [13]. Third, the anti-seizure medications (ASM) were a potential confounding factor. ASM could affect brain topology property and alertness [59, 65, 66]. In addition, it should be considered in this study that the effects of sedation by ASM might reduce alertness in this study. Fourth, heterogeneous seizure types in patients with TLE-non-FBTCS might also be a confounding factor on FC. The group with TLE-non-FBTCS that presented with a variety of seizure types, such as simple partial and/or focal complex seizures, were excluded from the FBTCS group. The focal complex seizures in TLE might have different patterns of impaired FC from the simple partial seizures in some brain areas [67].

## Conclusions

In this study, by using graph theory analysis of functional imaging, we found that TLE-FBTCS patients significantly higher small-worldness, normalized clustering coefficient (γ) and a trend of higher global network efficiency (gE) compared to TLE-non-FBTCS patients. That is, these groups have different patterns of the topological characteristics. The subgroup of RTLE-FBTCS patients had lower nodal efficiency in the left basal ganglia than HCs. We observed that the TLE-FBTCS had lower performance than those with TLE-non-FBTCS in intrinsic alertness, phasic alertness and alertness effect. This suggests that FBTCS might predispose a patient with TLE to show deterioration in alertness. We also found that the gE was negatively correlated with the mean RT in no cue trials in TLE-non-FBTCS patients, suggesting that the more serious the deficit is in intrinsic alertness, the lower the gE and the poorer the capability for information integration. The results of the current study need to be confirmed by future longitudinal and large sample size studies.

## Supplementary Information

**Additional file 1: Supplementary table**. Comparison of global parameters for patients and controls.

## Data Availability

The data are available upon request to the corresponding author.
