# Supplementary material for: A study of brain functional network and alertness changes in temporal lobe epilepsy with and without focal to bilateral tonic–clonic seizures
Source: BMC Neurol. 2022 Jan 7;22:14. doi: 10.1186/s12883-021-02525-w (PMC8740350; doi:10.1186/s12883-021-02525-w)
Supplement: Supplementary file 1 — Additional file 1: Supplementary table. Comparison of global parameters for patients and controls. [file 12883_2021_2525_MOESM1_ESM.docx]

| **Global**  **parameters 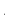** | **TLE-non-FBTCS** | | | **TLE- FBTCS** | | | **Health control** | | | **Statistical significance in ANCOVA F-value** | | |
| --- | --- | --- | --- | --- | --- | --- | --- | --- | --- | --- | --- | --- |
|  | **Left**  **(7)** | **Right**  **(11)** | **Unilateral (left or right)**  **(18)** | **Left**  **(10)** | **Right**  **(11)** | **Unilateral (left or right)**  **(21)** | **HC-1**  **(11)** | **HC-2**  **(11)** | **HC**  **(22)** | **Left** | **Right** | **Unilateral (left or right)** |
| **σ**  **(M±SD)** | 0.411±0.044 | 0.390±0.041 | 0.398±0.042 | 0.443±0.034 | 0.422±0.049 | 0.432±0.043 | 0.470±0.036 | 0.446±0.026 | 0.458±0.033 | / | 0.000 | 0.000 |
| **γ**  **(M±SD)** | 0.448±0.035 | 0.398±0.039 | 0.405±0.041 | 0.412±0.045 | 0.4284±0.050 | 0.437±0.043 | 0.477±0.038 | 0.451±0.027 | 0.463±0.034 | / | 0.000 | 0.000 |
| **λ**  **(M±SD)** | 0.335±0.002 | 0.337±0.004 | 0.336±0.003 | 0.333±0.001 | 0.334±0.002 | 0.334±0.002 | 0.334±0.001 | 0.333±0.002 | 0.334±0.002 | / | / | 0.005 |
| **gE**  **(M±SD)** | 0.216±0.003 | 0.212±0.006 | 0.216±0.003 | 0.218±0.001 | 0.216±0.004 | 0.213±0.005 | 0.217±0.001 | 0.218±0.001 | 0.218±0.001 | / | 0.016 | 0.001 |
| **locE**  **(M±SD)** | 0.256±0.007 | 0.256±0.008 | 0.254±0.005 | 0.253±0.004 | 0.255±0.005 | 0.256±0.008 | 0.255±0.006 | 0.251±0.006 | 0.253±0.006 | / | / | / |

Supplementary table

Comparison of global parameters for patients and controls.

σ, small-worldness; γ, normalized clustering coefficient; λ, normalized characteristic path length; gE, global network efficiency; locE, local network efficiency,

M±SD, mean ± standard deviation; ANOVA, one-way analysis of variance;
